# Metabolites from blood and formalin-fixed, paraffin-embedded tissue from participants with low- and high-grade prostate cancer: a pilot study

**DOI:** 10.3389/fonc.2026.1743012

**Published:** 2026-09-04

**Authors:** Rebecca E. Graff, Henrik L. Bengtsson, Jung H. Suh, Adam B. Olshen, Elizabeth Y. Wang, Ryan M. Allen, Erin L. Van Blarigan, Stacey A. Kenfield, Janet E. Cowan, Peter R. Carroll, Jeff Simko, June M. Chan

**Affiliations:** 1 Department of Epidemiology and Biostatistics, University of California, San Francisco, San Francisco, CA, United States; 2 Denali Therapeutics, San Francisco, CA, United States; 3 Helen Diller Family Comprehensive Cancer Center, University of California, San Francisco, San Francisco, CA, United States; 4 Department of Surgery, John A. Burns School of Medicine, University of Hawai`i at Manoa, Honolulu, HI, United States; 5 Loyola University Medical Center, Chicago, IL, United States; 6 Department of Urology, University of California, San Francisco, San Francisco, CA, United States; 7 Department of Pathology, University of California, San Francisco, San Francisco, CA, United States

**Keywords:** prostate cancer, metabolomics, Gleason score, plasma, FFPE

## Abstract

**Background:**

Identifying metabolites associated with prostate cancer (Pca) aggressiveness may elucidate mechanisms underlying disease severity. Doing so in plasma and formalin-fixed, paraffin-embedded (FFPE) tissue may accelerate discovery. In this cross-sectional pilot study, we generated hypotheses for further exploration by assessing the association between plasma metabolites and the Gleason score of individuals with Pca and by evaluating the correlation between plasma and FFPE metabolite levels.

**Methods:**

We examined plasma and FFPE samples from 10 individuals with Gleason score 7 (6 3 + 4, 4 4 + 3) and 9 individuals with Gleason score 9 (6 4 + 5, 3 5 + 4) tumors from a convenience sample of 19 men with Pca. We measured the relative abundance of polar metabolites at the time of radical prostatectomy. We used linear models of log_2_ fold changes to examine plasma metabolite levels relative to pathological tumor grade. Relationships among metabolite levels measured in plasma and FFPE tumor tissue within individuals across metabolites were examined using Pearson correlations.

**Results:**

Among the 18 selected plasma metabolites, which were chosen *a priori* because of their prior association with Pca aggressiveness, serine (*p* = 0.0051) and ornithine (*p* = 0.036) levels were higher in individuals with Gleason 9 than Gleason 7 Pca. After multiple testing correction, however, no associations were statistically significant. The median correlation between metabolite levels in plasma and FFPE tumor tissue was 0.45 (range: 0.40–0.53) for the 94 metabolites measured in both biospecimens.

**Conclusions:**

Plasma serine and ornithine levels demonstrated the largest differences between individuals with Gleason 7 and Gleason 9 Pca. Metabolite levels in FFPE prostate tissue samples were moderately correlated with plasma levels. Future studies with larger sample sizes are needed to further explore the hypotheses generated by this study.

## Introduction

Recent molecular and imaging tests have refined the management of prostate cancer (Pca) (1), but it remains challenging to distinguish low-risk from lethal disease at the time of diagnosis. The Gleason score is among the strongest predictors of disease progression (2), but its utility in localized disease can be limited; men who undergo active surveillance are monitored with serial biopsies that may miss high-grade foci of Pca (3–5). A non-invasive method to detect high-grade tumors may improve disease monitoring and inform treatment decisions.

One such method that may prove fruitful is the evaluation of circulating metabolites. Metabolomic data have the potential to enhance the recognition of aggressive Pca before it presents clinically. Emerging data have highlighted a number of intriguing metabolites and pathways in relation to Pca aggressiveness (6–9). These studies have done so using a variety of biospecimens, including blood, urine, and frozen or fresh prostate tissue. However, few studies have evaluated metabolites in formalin-fixed, paraffin-embedded (FFPE) prostate tumor samples (10–12), although such specimens are routinely collected during medical care for Pca and are stable over time. If it were possible to accurately assay FFPE tissue, then many more studies could evaluate the metabolomic basis of Pca. Furthermore, if circulating metabolite levels were reflective of the aggressiveness of the tissue metabolomic domain, then they could be leveraged to identify lethal disease early on, when interventions are more effective.

We conducted this pilot study of Pca metabolomics with two goals in mind: 1) to generate hypotheses about plasma metabolites associated with Gleason score at the time of radical prostatectomy (RP); and 2) to ascertain correlations between plasma, benign tissue, and tumor tissue metabolite levels both within individuals (across metabolites) and across individuals (for each metabolite).

## Methods

### Study population

The Department of Urology at the University of California, San Francisco (UCSF) maintains the Urologic Outcomes Database (UODB) (13), which houses clinicopathological, treatment, and outcome data for individuals with Pca undergoing active surveillance or RP. The UODB is linked to a biobank that preserves biological specimens from urologic oncology patients. As part of the standard biobanking protocol, men scheduled for RP are asked if they are willing to donate blood at the time of surgery. Consenting men provide blood just before surgery, under standard fasting conditions (minimum 8 h). Preoperative blood is drawn into ethylenediaminetetraacetic acid (EDTA) tubes, centrifuged for 20 min at 1,900 × *g*, and stored as aliquots of plasma, serum, and whole blood at -80 °C. RP tissue is fixed in formalin, serially sectioned at 4 mm thickness, and entirely embedded into FFPE tissue blocks. Each block is sectioned at 4 μm thickness and stained with hematoxylin and eosin (H&E) to identify tumor and normal areas by a board-certified pathologist (JS).

This pilot study leveraged a convenience sample of 19 men treated with RP who contributed matched preoperative plasma and both benign and tumor FFPE prostate samples. An additional five men contributed only matched benign and tumor FFPE prostate samples. For metabolomic profiling (described below), tumor and normal tissue samples were obtained from the FFPE sections using a 2 mm dermal punch. Gleason scores of Pca samples were determined by pathological review of RP tissue.

All participants consented to the collection of samples for research purposes. The study protocol was approved by the institutional review board at UCSF.

### Metabolomic profiling and processing

Polar metabolomic analyses of all plasma and FFPE samples were performed using liquid chromatography–tandem mass spectrometry (LC-MS/MS), as has been described in prior publications (10, 14–16). Metabolites with a signal-to-noise ratio < 5 or missing in ≥40% of the samples were omitted, after which peak intensities were normalized to the metabolite median and centered. Specifically, the 5%-trimmed sample mean was calculated on the logarithmic scale for each participant and then transformed back to the original scale. The intensities were subsequently rescaled so that all participants had the same target average, which was arbitrarily defined as the median signal of the first participant.

### Statistical analysis

To limit issues of multiple testing, we restricted the analysis of plasma metabolite levels in relation to Gleason score to a prespecified set of 18 metabolites selected through a qualitative review of the literature at the time the project was initiated, prioritizing metabolites with prior evidence of a relationship with Pca aggressiveness (15, 17–25). To compare metabolite levels between individuals with Gleason score 9 Pca and those with Gleason score 7 Pca, we implemented linear models using the limma R package (26). We specifically utilized the standard limma model for differential expression, which included an intercept and a term for the Gleason score. The moderated t-test was then used to identify differences in metabolite values between individuals with Gleason scores of 9 and 7. In sensitivity analyses, we compared individuals with 3 + 4 tumors to participants with 4 + 3 or 9 tumors.

Relationships among metabolite levels measured in plasma, benign prostate tissue, and tumor prostate tissue were examined using Pearson correlations, both within individuals across metabolites and across individuals for each metabolite.

All statistical analyses were carried out in R version 4.3.0.

## Results

Of the 19 participants with available plasma, 10 had an RP Gleason score of 7 (6 with 3 + 4, 4 with 4 + 3), and 9 had an RP Gleason score of 9 (6 with 4 + 5, 3 with 5 + 4) (**Table 1**). The median age of the participants with available plasma at RP was 64 years (interquartile range: 11.5 years).

**Table 1:** Characteristics of 19 participants with both plasma and FFPE tissue and of five participants with FFPE tissue only.

ID	Gleason score	Age at RP	Tumor tissue site	Tumor %	Normal tissue site	Normal %
Plasma and FFPE						
001	4 + 5	71	Bilateral posterior mid	90%	Left posterolateral mid	10%
002	5 + 4	70	Right posterolateral mid	95%	Left lateral mid	10%
003	4 + 3	71	Right posterior mid	80%	Left anterior mid	20%
004	5 + 4	62	Bilateral posterior mid	80%	Right anterior mid	20%
005	4 + 5	68	Bilateral posterior apex	70%	Left lateral mid	10%
006	4 + 3	55	Bilateral posterior mid	70%	Right anterior mid	20%
007	4 + 5	54	Bilateral posterior apex	90%	Right anterior apex	20%
008	3 + 4	48	Bilateral posterior apex	70%	Left anterior base	40%
009	4 + 3	69	Bilateral posterior apex	90%	Left lateral mid	20%
010	4 + 5	64	Left lateral mid	95%	Right lateral mid	30%
011	3 + 4	58	Right posterior apex	90%	Left lateral base	30%
012	3 + 4	59	Left anterior mid	90%	Right anterior base	20%
013	5 + 4	61	Left lateral mid	90%	Left posterior mid	40%
014	3 + 4	71	Left posterolateral mid	80%	Right posterolateral base	30%
015	4 + 3	67	Left lateral apex	90%	Right posterolateral mid	40%
016	4 + 5	64	Left anterior apex	95%	Bilateral posterior mid	60%
017	4 + 5	54	Left posterolateral base	80%	Left posterior mid	50%
018	3 + 4	53	Left posterolateral mid	90%	Right lateral mid	30%
019	3 + 4	67	Bilateral central apex	70%	Right posterolateral base	30%
FFPE only						
020	5 + 4	70	Right lateral base	70%	Right posterolateral base	30%
021	3 + 4	67	Right lateral and anterior mid	70%	Left posterior mid	40%
022	4 + 5	78	Left lateral apex	95%	Right lateral apex	30%
023	4 + 3	70	Right lateral posterior mid	90%	Bilateral anterior base	30%
024	3 + 4	71	Posterior mid	80%	Left anterior apex	40%

### Associations between plasma metabolites and Gleason score

**Figure 1:**
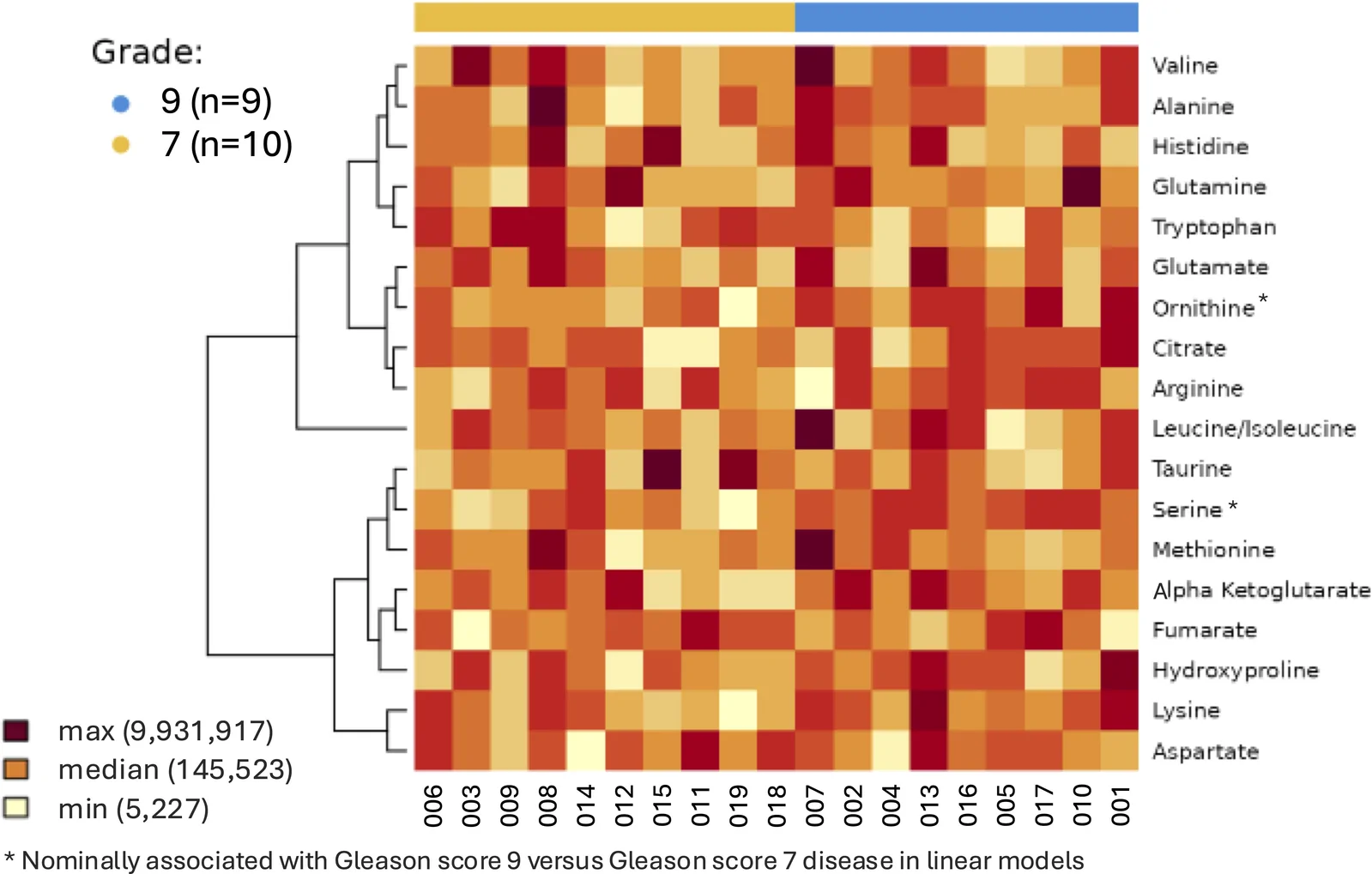
Heatmap and hierarchical clustering of log_2_ levels of 18 plasma metabolites of interest annotated by Gleason 7 (n = 10) versus 9 (n = 9) prostate cancer in a total of 19 participants. Rows were centered and scaled. Distances were computed using the Euclidean distance method, and hierarchical clustering was performed using the complete linkage method. The maximum, median, and minimum values are specified for all 18 metabolites on the original scale (i.e., not log_2_).

**Figure 1** depicts a heatmap and hierarchical clustering of the 18 metabolites of interest according to Gleason scores 7 versus 9 disease. No clear patterns emerged, and none of the metabolites were statistically significantly associated with the Gleason score following correction for multiple testing using the Benjamini-Hochberg method (**Table 2**). There were, however, two metabolites that were nominally associated with Gleason score based on linear models; higher levels of plasma serine (*p* = 0.0051) and ornithine (*p* = 0.036) were associated with Gleason 9 disease. Results for the remaining 98 metabolites successfully measured in plasma are presented in **Supplementary Table 1**. An additional three metabolites (2-aminooctanoic acid, acetyl-lysine, and sedoheptulose-1,7,-bisphosphate) demonstrated nominal evidence of an association.

**Table 2:** Associations between 18 plasma metabolites of interest and Gleason 7 (n=10) versus 9 (n=9) prostate cancer in the 19 participants.

Metabolite	Gleason score 7 median level^a^	Gleason score 9 median level^a^	Fold change (95% CI)^b^	*p*-value^b^	Adjusted *p*-value^b^^,^^c^
Serine	58588	82860	1.32 (1.10, 1.59)	0.0051	0.092
Ornithine	194391	203889	1.24 (1.02, 1.50)	0.036	0.32
Lysine	12771	14302	1.15 (0.96, 1.38)	0.12	0.64
Hydroxyproline	25016	52760	1.43 (0.88, 2.31)	0.14	0.64
Tryptophan	748070	610330	0.87 (0.69, 1.10)	0.23	0.75
alpha-ketoglutarate	25197	30336	1.10 (0.93, 1.29)	0.27	0.75
Glutamine	867248	1015170	1.11 (0.90, 1.38)	0.30	0.75
Citrate	179618	198796	1.14 (0.86, 1.52)	0.34	0.75
Alanine	452527	638286	1.13 (0.85, 1.51)	0.38	0.75
Arginine	137217	150190	1.07 (0.87, 1.32)	0.52	0.88
Methionine	44406	55011	1.09 (0.80, 1.49)	0.58	0.88
Fumarate	23687	24822	0.94 (0.73, 1.20)	0.59	0.88
Taurine	83150	77343	0.96 (0.77, 1.19)	0.67	0.92
Leucine/Isoleucine	4528074	5004493	1.04 (0.80, 1.35)	0.75	0.94
Aspartate	7431	7017	0.98 (0.80, 1.20)	0.81	0.94
Valine	607891	730672	1.02 (0.79, 1.32)	0.88	0.94
Glutamate	215020	184608	1.02 (0.77, 1.34)	0.89	0.94
Histidine	326406	363683	0.99 (0.84, 1.18)	0.94	0.94

CI – confidence interval.

^a^ Based on the raw data.

^b^ From linear models of log_2_ metabolite levels.

^c^ Adjusted according to the Benjamini-Hochberg procedure.

Sensitivity analyses that compared levels of the 18 metabolites of interest between the 6 individuals with Gleason 3 + 4 Pca and the 13 participants with higher-grade disease were similarly not statistically significant (**Supplementary Figure 1**; **Supplementary Table 2**). Only ornithine (*p* = 0.040) was associated with higher-grade disease at a nominal significance threshold. Among the remaining 98 metabolites measured in plasma, only N6-acetyl-L-lysine and acetyl-lysine demonstrated nominal evidence of an association (**Supplementary Table 3**).

### Correlations among plasma, benign tissue, and Pca tissue metabolites

**Figure 2:**
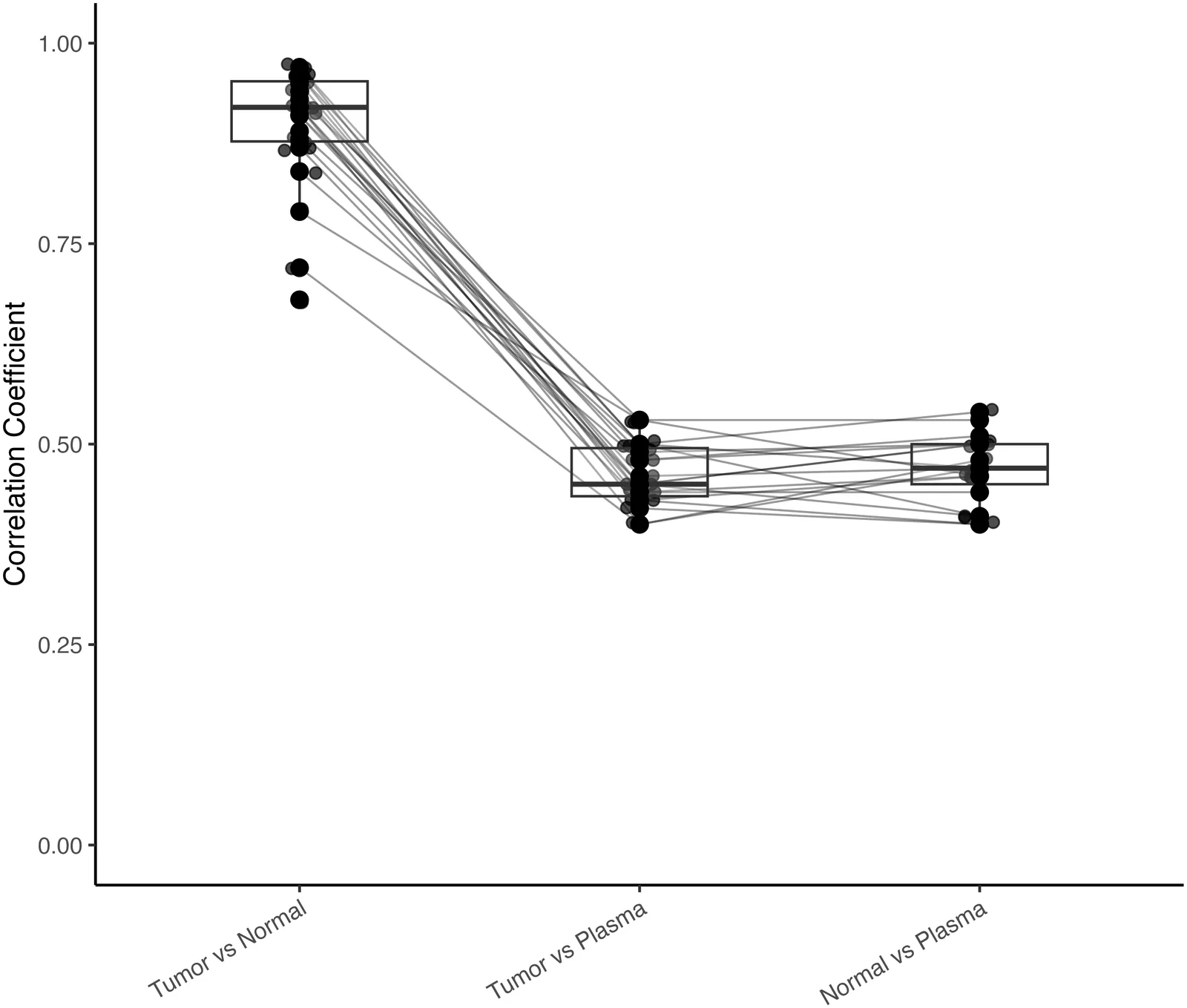
Correlations between plasma, benign tissue, and tumor tissue metabolite levels within individuals across metabolites. Each point represents the correlation across metabolites for a single individual for the specified comparison, with lines connecting values from the same individual. Boxplots indicate the median and interquartile range for each comparison.

Of the 116 metabolites that were successfully measured in plasma, 94 (81%) were also measured in the FFPE samples (**Supplementary Table 4**). Within individuals, correlations were generally stronger between benign and tumor tissue levels than between either tissue measurement and plasma levels (**Figure 2**; **Supplementary Table 5**). Among the 24 individuals with metabolite levels in both benign and tumor tissues, the median correlation was 0.92 (range: 0.68–0.97). Among 19 individuals with available tissue and plasma metabolite levels, the median correlation between benign tissue and plasma levels was 0.47 (range: 0.40–0.54). The median correlation between tumor tissue and plasma metabolite levels was 0.45 (range: 0.40–0.53).

**Figure 3:**
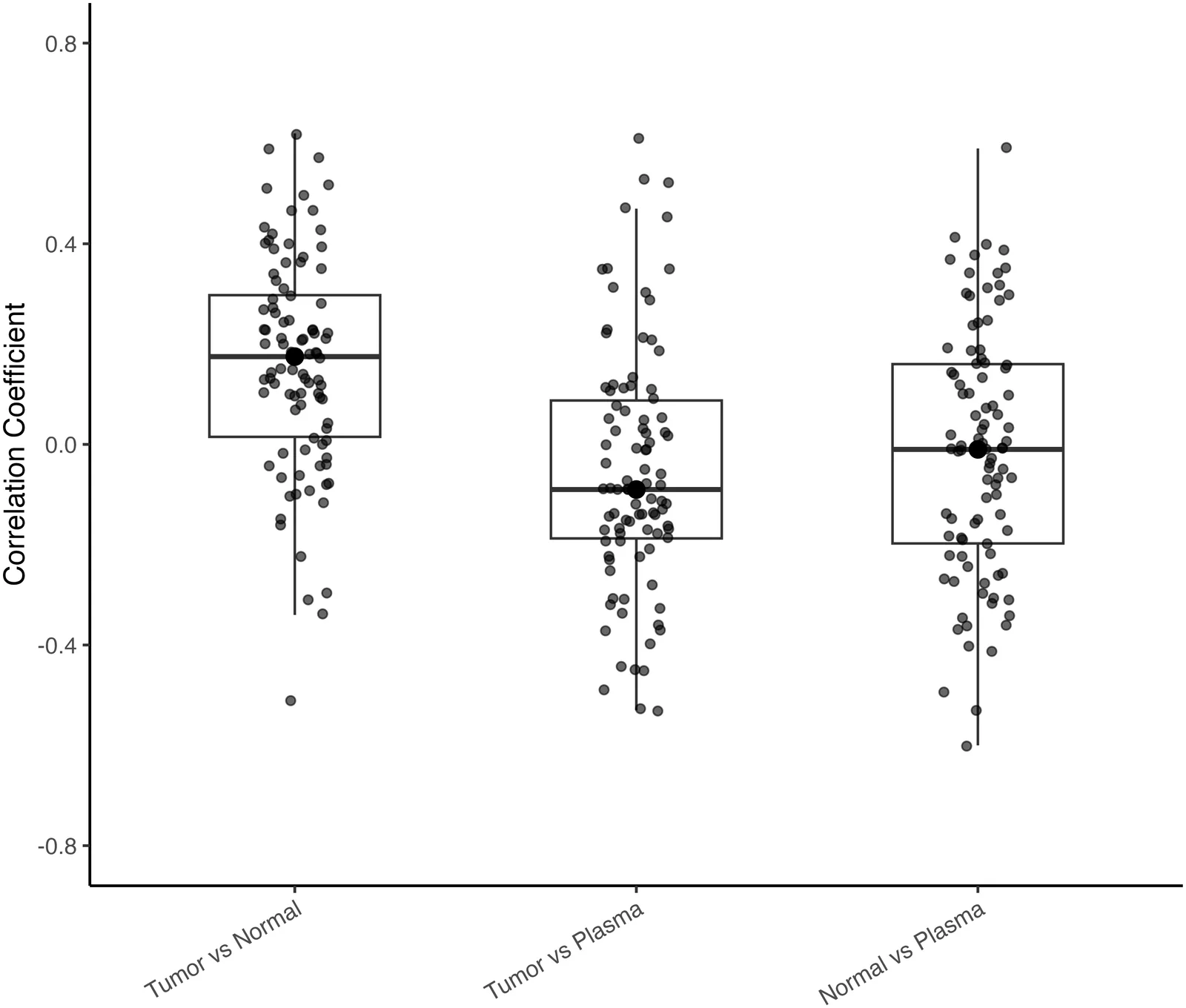
Distribution of correlations between plasma, benign tissue, and tumor tissue metabolite levels across individuals. Each point represents a single metabolite, and boxplots indicate the median and interquartile range for each comparison.

Analyses of metabolite levels across individuals for each metabolite revealed that correlations between benign and tumor tissue were positive for 72 out of 94 metabolites (77%) and generally moderate in magnitude (median: 0.17) (**Figure 3**; **Supplementary Table 6**). Correlations between tumor tissue and plasma levels were more variable and frequently inverse (median: -0.09), whereas correlations between benign tissue and plasma levels were generally weaker and closer to null (median: -0.01). The metabolites that were most strongly positively correlated between tumor and plasma levels were urea (r^2^: 0.61), methylnicotinamide (r^2^: 0.53), and hypoxanthine (r^2^: 0.52).

## Discussion

In this pilot study of Pca metabolomics, we observed largely null associations between circulating metabolites and Gleason score. There was, however, suggestive evidence that circulating serine and ornithine may be associated with tumor Gleason score. We also achieved metabolite measurements in FFPE tissue, and these values were moderately correlated with circulating metabolite levels within individuals. Metabolite levels in benign and tumor FFPE tissue were more strongly correlated. Across individuals, correlations between tissue and plasma levels of individual metabolites were variable and often modest.

Although the results of this small pilot study should be treated as exploratory, mechanistic evidence suggests that both serine and ornithine may contribute to Pca aggressiveness. Serine synthesis provides intermediates to the Krebs cycle, which sustains cancer metabolism (27). In addition, cells lacking p53—a tumor suppressor gene that is often mutated in Pca—may respond to serine-depletion therapy (27, 28); increased serine would presumably work antagonistically. Ornithine may plausibly promote Pca aggressiveness via its role in the synthesis of polyamines, which are involved in tumor growth, invasion, and development of metastases (29, 30). In addition, ornithine is central to the urea cycle, dysregulation of which has also been implicated in prostate and other cancers (31, 32). Both serine and ornithine are components of the glycine, serine, and threonine metabolism pathway, which refuels one-carbon metabolism, the process that cycles carbon units from amino acids to produce proteins, lipids, and nucleic acids essential to cell growth and proliferation (27, 33). This pathway also plays an important role in cellular redox balance, fuels heme biosynthesis, and sustains oxidative phosphorylation (34), thereby increasing the proliferation rate of cancer cells (33, 35, 36). These pathway-level considerations should be interpreted in the context of largely null associations across individual metabolites in this study.

Preliminary data from other studies point to aberrant mobilization of glycine, serine, and threonine metabolism in aggressive Pca (11, 15, 17–19, 24, 25, 29, 37–61). With respect to the Gleason score specifically, one prior study identified urinary upregulation of the pathway as a whole in individuals with more poorly differentiated tumors (45). In another study, higher serine levels in seminal plasma were associated with a higher Gleason score (38). While previous studies have not identified associations between ornithine and tumor grade, several have shown positive associations with other Pca characteristics indicative of aggressiveness (e.g., the development of biochemical recurrence or metastases) (17, 29, 49, 58). Our results are thus consistent with the existing literature and mechanistically plausible, although they should be interpreted cautiously given the small sample size and only nominally significant p-values. We also did not observe consistent differences in other metabolites within related pathways, including those involved in glycine/serine/one-carbon metabolism (e.g., 3-phospho-serine, methionine, threonine) and the urea cycle and polyamine synthesis (e.g., arginine, citrulline, urea, putrescine). This lack of concordant changes across metabolically linked compounds suggests that any alterations in these pathways may be modest or localized rather than reflected by coordinated shifts across multiple metabolites.

FFPE tissue specimens are not often leveraged for studies of metabolomics due to concerns about changes in metabolites during preservation. Nevertheless, preliminary studies support the feasibility and utility of mass spectrometry-based metabolic profiling of FFPE tissue for Pca (10–12) and other tumor types (62–71). Those comparing metabolite levels in fresh-frozen versus FFPE prostate tumor tissue indicate that the latter have reduced coverage but can still recapitulate key biological states (10, 11). Our study adds to the limited existing body of evidence that FFPE prostate tissue can yield useful biochemical information.

The inclusion of matched benign and tumor tissue from the same prostate enabled within-individual comparisons that reduced inter-individual variability. Within individuals, metabolite profiles were highly concordant between benign and tumor FFPE tissue, suggesting the preservation of biologically meaningful metabolic patterns despite fixation and the presence of shared metabolic features within the prostate microenvironment. Correlations between tissue and plasma metabolite levels were more modest, indicating that circulating metabolites only partially reflect tissue metabolic profiles. In analyses across individuals for individual metabolites, concordance between plasma and tissue levels was variable, with only a subset of metabolites demonstrating moderate cross-matrix correlations. Together, these findings indicate that FFPE-derived metabolite measurements preserve biologically meaningful metabolic relationships within prostate tissue, while plasma metabolites provide a more distal and less specific reflection of tumor metabolism. Given that FFPE tissue is more commonly available than other tissue types among individuals with long-term follow-up, metabolomics research leveraging FFPE samples has the potential to accelerate the identification of biochemicals that indicate Pca aggressiveness.

Because our study was intended as a pilot metabolomic analysis of samples from the UCSF urologic oncology biobank, its sample size was limited for meaningful biological inference. This limitation is particularly relevant in metabolomics, where effect sizes are often small and distributed across correlated pathways. We were unable to adjust for secondary variables that would have strengthened causal interpretation. We nonetheless demonstrated the utility of our plasma and FFPE samples for future metabolomics research.

Taken together, the largely null associations between individual circulating metabolites and Gleason score suggest that metabolic differences in plasma related to tumor aggressiveness may be modest in magnitude or distributed across multiple interconnected pathways rather than driven by large changes in individual metabolites. In this context, serine and ornithine emerged as candidates for subsequent investigation for Pca aggressiveness. We also demonstrated that metabolites are detectable in FFPE tissue samples, which may prove useful for the future discovery of Pca biomarkers. Such studies should include larger sample sizes and implement comprehensive covariate adjustment to disentangle systemic metabolic influences from tumor-specific biology.

## Data Availability

The datasets presented in this article are not publicly available because the original research consent form stated that data would only be reported in grouped de-identified form. Requests to access the datasets should be directed to the corresponding author.
